# Temperature adaptation in structure and function in lactate dehydrogenase-A reflects convergent evolution in a few key protein regions

**DOI:** 10.1073/pnas.2517759122

**Published:** 2025-10-10

**Authors:** Xiao-Lu Zhu, Ming-Ling Liao, Lin-Xuan Ma, George N. Somero, Yun-Wei Dong

**Affiliations:** ^a^The Key Laboratory of Mariculture, Ministry of Education, Fisheries College, Ocean University of China, Qingdao 266003, China; ^b^Shandong Key Laboratory of Green Mariculture and Smart Fishery, Fisheries College, Ocean University of China, Qingdao 266003, China; ^c^Academy of Future Ocean, Ocean University of China, Qingdao 266100, China; ^d^Department of Biology, Hopkins Marine Station, Stanford University, Pacific Grove, CA 93950

**Keywords:** convergent evolution, enzyme structure, lactate dehydrogenase-A, marine fish, thermal adaptation–related sequence sites

## Abstract

Adaptive differences in the thermal stabilities of enzyme structure and function play critical roles in establishing the thermal optima and limits of all organisms. Thus, understanding the mechanisms underlying these adaptations can yield insights into protein structure–function relationships, protein evolution, and the consequences of temperature shifts on species distributions. Past studies have suggested that only a small number of amino acid substitutions are needed for adaptive change, but whether similar sites in the sequence, which we term thermal adaptation–related sequence sites (TRSS), and similar changes in amino acid content at these TRSS occur across widely different taxa remains to be elucidated. For detecting TRSS among orthologs of species adapted to a wide range of temperatures, we investigated 277 lactate dehydrogenase-A (LDH-A) orthologs in marine fish from diverse habitats. We validated the importance of several TRSS variants using site-directed mutagenesis on zebrafish LDH-A, effectively recreating the variants in the zebrafish ortholog. Our results indicate that enzyme thermal adaptation arises primarily from a few substitutions that influence hydrophobicity in functionally important regions of secondary structures. These findings reveal striking convergence in the sites (the TRSS) of temperature-adaptive evolution of LDH-As and provide insights into the types of amino acid substitutions that foster adaptation to temperature. Furthermore, the patterns of convergent evolution identified in this study supported development of a deep learning model for predicting thermal limits. This model can provide an important tool for predicting thermal ranges of species and the potential effects of temperature change on distribution patterns.

## Significance

Substitutions in a few amino acids can significantly alter the structural and functional responses of enzymes to temperature, traits that are closely related to establishing the thermal optima and limits of organisms. A cross-taxa analysis of 277 fish lactate dehydrogenase-A (LDH-A) orthologs, which incorporated bioinformatic, in silico and in vitro methodologies, reveals striking convergence in the sites of temperature-adaptive evolution of LDH-As. Based on these findings, a deep learning model was developed to predict thermal limits of fish. These results further the understanding of how fish adapt to divergent thermal environments and provide a valuable model for assessing the potential thermal ranges of fish.

The essential role of temperature-adaptive differences in enzymes of species evolving in different thermal environments has long been a focus of studies of biochemical evolution (1–4). Numerous structural and functional properties of enzymes are closely related to the temperatures at which they have evolved (5–7). Catalytic activities and ligand (substrate and cofactor) binding abilities, which are both highly sensitive to acute changes in temperature, are highly conserved in differently thermally adapted species at their respective habitat temperatures (8). The structural bases of these adaptations in function have been partially resolved. Commonly, only one to a few changes in amino acid sequence are sufficient to adaptively modify the thermal responses of enzymes; these adaptive changes often lead to localized changes in protein flexibility (5, 8–11).

Although these earlier studies, which involved a relatively small number of species, have clearly shown the importance of temperature-adaptive amino acid substitutions and provided insights into how these changes in structure lead to adaptive change in function, the studies’ limitations in taxonomic scope have precluded determining whether broad, cross-taxa patterns of adaptation exist. This lack of knowledge about cross-taxa adaptive patterns is a consequence of several limitations faced in earlier work. In particular, the relative scarcity of available sequence data on which to base comparisons and the lack of powerful bioinformatic methods to detect patterns in these large datasets limited the scope of earlier analyses. Here, we sample a wide range (277 species) of marine fish from widely different habitats and employ contemporary molecular, in silico, bioinformatic, and deep learning approaches to clarify whether a common set of adaptively important sites in the protein’s sequence, which we term thermal adaptation–related sequence sites (TRSS) can be detected and whether at these sites particular types of amino acids are employed in adaptation to either high- or low temperatures. Thus, we initially identify TRSS and then explore their functional significance using a range of techniques, ranging from in silico simulation of structural flexibility to site-directed mutagenesis (SDM) to generate variant forms in which the TRSS have been altered. We then briefly explore the issue of whether the thermal limits and distributions of species can be predicted by these TRSS. We basically inquire whether certain types of amino acid substitutions at a relatively small number of TRSS allow characterization of macroscopic traits like organismal thermal optima and thermal limits.

The approaches we employ allow a variety of questions about evolution and biogeographic distributions to be explored. For example, are the temperature-adaptive enzyme thermal phenotypes a reflection of phylogenetic history or do they reflect convergent evolution (12–14)? Our current knowledge of adaptive change across taxa suggests that convergent evolution is likely to be prevalent (10, 15), but broad generalizations are difficult to support without more extensive analysis of sequence differences among orthologs of many species with different evolutionary thermal histories. Thus, with large-scale assessments across many taxa, it may be possible to characterize in a statistically powerful manner the TRSS from hundreds of species with long divergence times to further delineate the importance of convergent evolution. Characterizing the occurrence of convergent evolution of enzyme thermal adaptation will provide additional insights into whether species can respond to changes in environmental temperature in a predictable manner (16).

The analysis of these and other broad-scale questions benefits from newly developed protocols in deep learning (DL). These approaches make large-scale simulation possible for a number of phenomena, including protein three-dimensional (3D) structural prediction and biochemical mechanisms of enzyme thermal adaptation (17, 18). For example, by using *ColabFold*, the mechanistic linkage between enzyme thermal adaptation and 3D structural variations caused by a few amino acid changes could be detected among hundreds of teleosts (19). There is increasing evidence for common mechanistic determinants in enzyme thermal adaptation (8), and the occurrence of these mechanistic similarities can be well captured by DL models (20).

In the present study, we performed an analysis involving hundreds of orthologs of the glycolytic enzyme lactate dehydrogenase-A (LDH-A (EC 1.1.1.27) from marine fish, a taxonomically diverse group in which LDH-A has received extensive study (5, 8, 9, 21, 22). LDH evolves slowly and is highly conserved from bacteria to vertebrates (3, 5, 23), making it a powerful study system for investigating temperature-adaptive molecular evolution, as shown by studies that span more than a half-century (1). Additionally, the catalytic mechanism and 3D structure of LDH-A have received detailed study, making it an ideal system for linking amino acid substitutions to adaptive changes in stability and function (3, 9, 24).

To expand our knowledge of LDH-A evolution and structure–function relationships, an accelerated *AlphaFold*-based workflow, *ColabFold*, was used to predict the 3D structures of LDH-As and thereby identify sites in the folded enzyme structure where amino acid substitutions occurred. The potentially temperature-adaptive roles of amino acid substitutions at TRSS were examined using both in silico and in vitro approaches. For in silico studies, molecular dynamics simulations (*Gromacs*) were used to quantify the flexibility of LDH-A structures that play critical roles in function and protein stability. The conclusions from the in silico studies of TRSS were tested empirically through in vitro SDM experiments using LDH-A of the genomically well-characterized “model” fish, the zebrafish *Danio rerio.* With the TRSS for which an adaptive role was validated using SDM and in vitro studies of stability and kinetic function, a graph neural network (GNN) and gradient boosting model (XGBoost) framework was built and trained to predict species’ thermal limits from this biochemical information. Our results provide deeper insights into how amino acid substitutions achieve enzyme thermal adaptation and show how common convergent evolution at a few sites in the protein sequence is in achieving these adaptations. Using these data, we develop a DL model that provides an effective biochemistry-based tool for predicting species thermal tolerance.

## Results

### LDH-A Sequences in Marine Fish Adapted to Widely Different Temperatures.

**Fig. 1.**
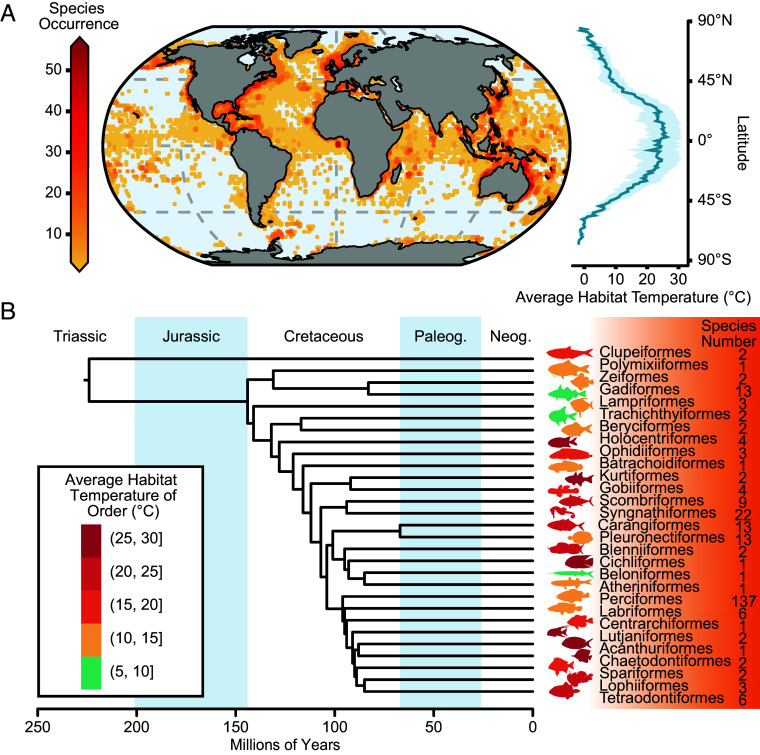
The variety of marine fish used in this study and their global distributions. (*A*) Geographical map at 2 × 2 arcdegree resolution. Color (coded by vertical bar at *Left* of figure) represents the number of species occurrence records in each grid. Average habitat temperatures (± SD) across latitude are shown in the line graph. (*B*) Phylogeny of fish used in the present study. Orders of fishes in this research were distinguished and extracted from an evolutionary tree of Actinopterygii (*Materials and Methods*). Colors of the cartoon silhouettes divide orders into five thermal property groups (*Inset* to figure). Species numbers are shown following each order.

A total of 277 fish LDH-A orthologs and relevant habitat temperature data were used to generate information on adaptive variation in amino acid sequences of LDH-A of marine fish from habitats that ranged from polar to equatorial waters and whose temperatures ranged from −1.6 °C to 29.5 °C (Fig. 1*A*).

These fishes belonged to 29 orders (excluding 16 species without precise order records), with a divergence time extending back as far as the Triassic period (over 200 MA) (Fig. 1*B*). Notably, lineages with close evolutionary affinities may have strikingly different thermal environments (body temperatures), which facilitates the ability to attribute sequence differences to effects of adaptation temperature rather than phylogeny per se. For example, although the Kurtiformes and Gobiiformes are sister groups, they live in environments where average habitat temperatures are 27.42 °C and 16.65 °C, respectively.

**Fig. 2.**
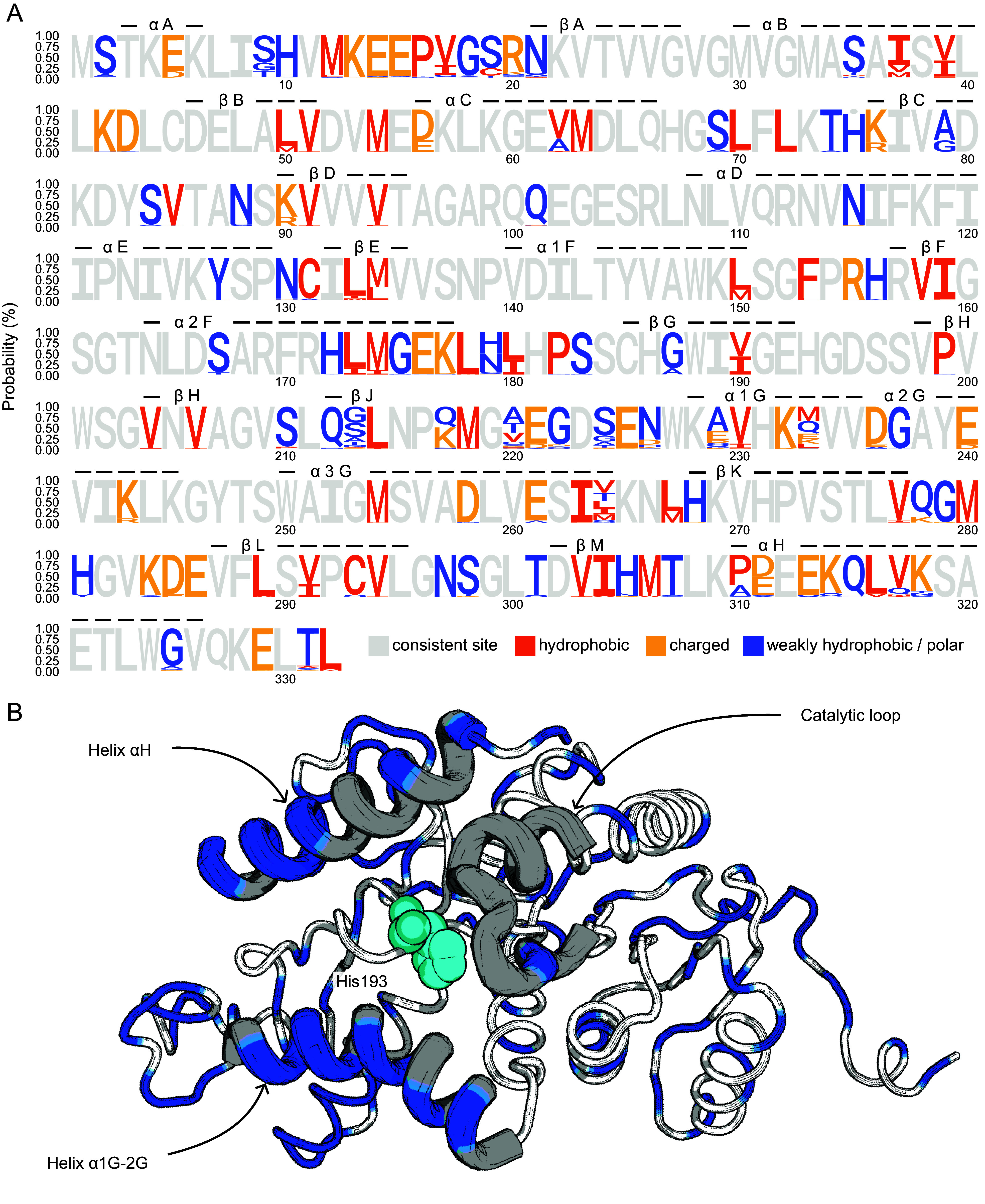
Sequence alignment of LDH-A orthologs of marine fish. (*A*) Relative frequency of amino acids in 277 aligned LDH-A orthologs. The dot at site 75 represents the gap in the alignment of LDH-A sequences. Consistent sites were colored with light gray, and substitutions were highlighted with brighter colors that indicate the three amino acid categories: charged (DEKR), hydrophobic (LVWIFMPC), or weakly hydrophobic/polar (AGNQSTHY) (25). Secondary structures are shown in the top line. (*B*) Simulated 3D structure of LDH-A monomer. The space-filled His193 indicates the region of the catalytic active site. Three mobile regions (MRs) are shown in dark gray and blue using lines with a thicker structure; blue sites represent the 3D position where substitutions were observed.

The ortholog sequence data we gathered from genomic analysis or obtained from earlier studies of LDH-A revealed the enzyme sequence to be highly conserved, as expected. Most species had 332-residue LDH-As, except for 29 notothenioid species that contained a deletion at the position of His75. In total, 127 substitution sites, i.e., sites where sequences differed between species, were detected and found to be distributed at numerous positions in the sequence across all 277 fish orthologs (Fig. 2*A*).

Although amino acid substitutions occurred at numerous sites along the sequence, they were not distributed randomly. As would be predicted, no substitutions occurred in the substrate and cofactor binding sites. These conservative properties reflect the mechanism of LDH function. In particular, in all LDH-A orthologs, active site His193 acts as a proton acceptor and donor and is considered essential for binding and catalysis, with Arg106 and Arg169 serving as the electrostatic stabilizers. Asp166, which serves as a reaction enhancer to form a dyad with His193, is also conserved. Thus, adaptive changes in LDH-A function do not arise from changes in the amino acids that directly interact with substrate and cofactor.

Substitutions commonly were found in the mobile regions (MRs) that are involved in the conformational changes that are essential for the binding and catalytic functions of the enzyme. These MRs include part of helix αD along with helices α1G-2G and segments around helix αH (Fig. 2*B*). These MRs move during catalysis to, first, allow passage of NADH and pyruvate to the active site. Second, movements that close these three MRs together lead to the formation of a catalytic vacuole that is isolated from the surrounding medium and functions as the site where substrate is converted to product. Following this conversion, these mobile structures must reopen to release lactate and NAD^+^ (or, in the reverse reaction, pyruvate and NADH). A total of 19 substitutions occurred in MRs among the total 127 substitutions detected. The functional significance of these MR-localized substitutions is treated below.

**Fig. 3.**
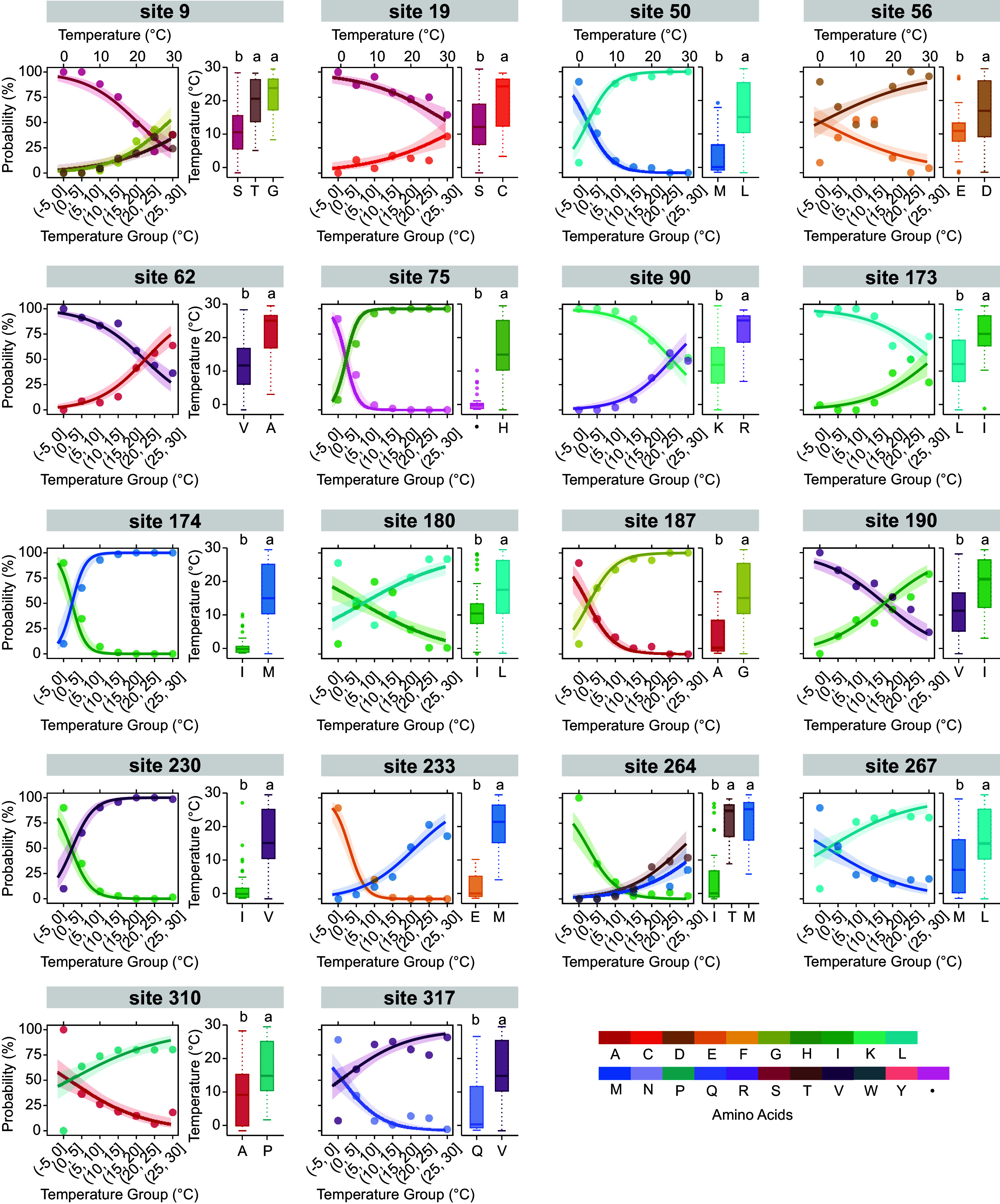
Detection of thermal-related sequence sites (TRSS). Binomial regression and significance tests between substitutions/variants and species average habitat temperature are shown in the line graph and box graph, respectively. The same letters in the box graph annotated at the top indicate there was no significant difference between the variants. The point graph represents the probability of each amino acid in the different temperature groups, determined by the species’ average habitat temperature.

In order to identify the TRSS found among the full set of 127 substitution sites, a rigorous protocol that included binomial regression and significance tests (ANOVA or Kruskal–Wallis test) between variants and habitat temperature was designed (*SI Appendix*, Fig. S1*A*). After performing the binomial regression and significance tests, only 18 substitution sites satisfied our criteria for TRSS (*Materials and Methods*), including four in the MRs. In the case of the statistical relationship between thermal adaptation and amino acid composition, isoleucine (Ile) was mostly used for cold-adaptation (22% TRSS), while leucine (Leu) and methionine (Met) were mostly used for warm-adaptation (17% TRSS) (Fig. 3). However, the same amino acids in different locations may respond oppositely in thermal adaptation, such as isoleucine and valine in sites 190 and 230. This variation in thermal adaptation of the same type of residue in different locations reflects the fact that the effects of a specific type of residue on protein stability and function not only depend on its inherent physicochemical properties but also depend strongly on properties of neighboring residues, such as their size, polarity, charge, and hydrophobicity.

### Structural Stability and Kinetics.

**Fig. 4.**
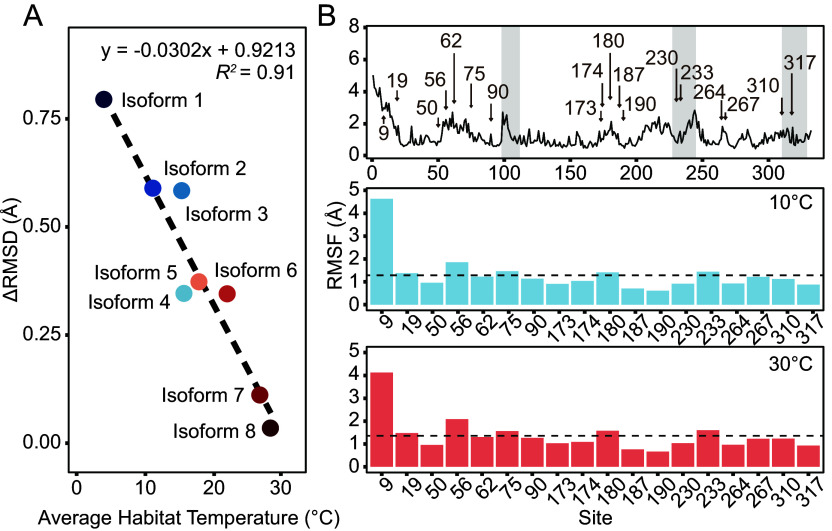
Molecular dynamic simulation results of LDH-A. (*A*) The ΔRMSD (the amount of increase in the RMSD value between 10 and 30 °C) over the equilibration state (10 to 20 ns) correlates with species’ average habitat temperature for 8 isoforms of LDH-A (Dataset S1). (*B*) The rms fluctuation (RMSF) for individual residues over the equilibration state (10 to 20 ns) at simulated temperatures of 10 and 30 °C of eight isoforms. The *Top* plot showed the distribution of TRSS across the LDH-As. Gray background regions represented the MRs. Brown numbers with arrows point to the TRSS. The *Middle* plot shows the average residue-specific RMSF of TRSS among eight isoforms simulated at 10 °C, with the dashed line representing the average RMSF across all sites of the eight isoforms. The *Bottom* plot shows the average residue-specific RMSF of TRSS among eight isoforms simulated at 30 °C, with the dashed line representing the average RMSF across all sites of the eight isoforms.

The structural stabilities of eight LDH-A isoforms from species spanning a wide evolutionary thermal history range (3.73 °C to 28.26 °C, see *SI Appendix*, Table S1) were investigated using molecular dynamic simulations to determine the general pattern between the whole sequence structural stability and the enzymatic thermal adaptation. Across all eight isoforms there was a decrease in ΔRMSD (the amount of increase in the RMSD value between 10 and 30 °C) with an increase in average habitat (adaptation) temperature (*SI Appendix*, Fig. S2 and Fig. 4*A*, *R^2^* = 0.91), indicating that the stiffness of the peptide backbone increases with adaptation temperature. In addition, the rms fluctuation (RMSF) values differed among regions of the proteins in accord with the roles of flexible regions in the catalytic process. Higher local RMSFs reflected the movements of residues in the three MRs (MRs, i.e., catalytic loop, helices α1G-2G, and helix αH) (Fig. 4*B*). It is noteworthy that 18 TRSS were distributed among the more flexible regions of the enzyme. Furthermore, four TRSS (230#, 233#, 310#, and 317#) belong to MRs, and two (173# and 174#) belong to the secondary structure of the active site (α2F). Given the essential role of active sites in enzymatic reactions, sequence variation near these sites has the potential to alter enzyme activity, even if the residues in the active site are unchanged. In addition to influencing structural stability, such variants can affect enzyme function by affecting substrate access through stereospecific blockade.

**Fig. 5.**
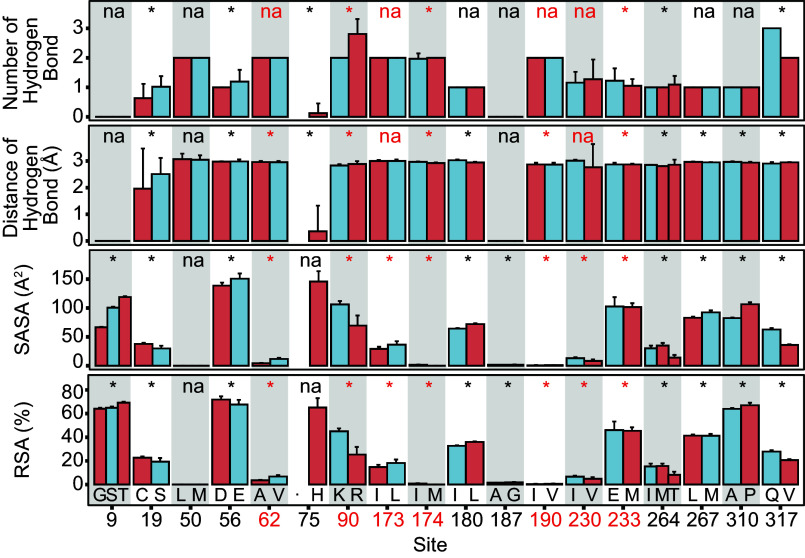
Structural features of LDH-A. The number of hydrogen bonds, distance of hydrogen bonds, SASA, and RSA of TRSS. The color blue represents the cold-adapted amino acid type, while the color red indicates the warm-adapted amino acid type. Results of the significance test (α = 0.05) are given at the top of each site. The dot at site 75 represents the aligned gap of LDH-As. Sites in red color were validated in a SDM experiment.

To understand the relationship between structural features of TRSS and temperature adaptation, the number of hydrogen bonds, distances of hydrogen bonds, solvent accessible surface area (SASA), and relative accessible surface area (RSA) were compared between different variants of each TRSS (Fig. 5). All of these features would be predicted to show a highly consistent temperature-dependent pattern. For the number of hydrogen bonds, a total of eight TRSS showed significant differences between cold-adapted variant type and warm-adapted variant type. Four TRSS showed the characteristics of a significantly higher number of hydrogen bonds of cold-adapted variant type rather than warm-adapted variant type. In case of the distance of hydrogen bonds, a total of thirteen TRSS showed significant differences between cold-adapted variant type and warm-adapted variant type. Of these thirteen TRSS, six showed the characteristics of significantly longer distance of hydrogen bonds found with the cold-adapted variant type rather than the warm-adapted variant type. SASA and RSA were used to measure the hydrophobicity characteristics of TRSS. Both SASA and RSA reflect the solvent exposure of amino acid residues and are associated with protein hydrophobicity (26). Among the 18 TRSS, 16 TRSS showed significant differences between the cold-adapted variant type and the warm-adapted variant type in both the SASA and RSA estimations: 11 TRSS in SASA and 9 TRSS in RSA showed significantly lower hydrophobicity (i.e., a larger value of SASA or RSA) for the cold-adapted variant type compared to the warm-adapted variant type.

**Fig. 6.**
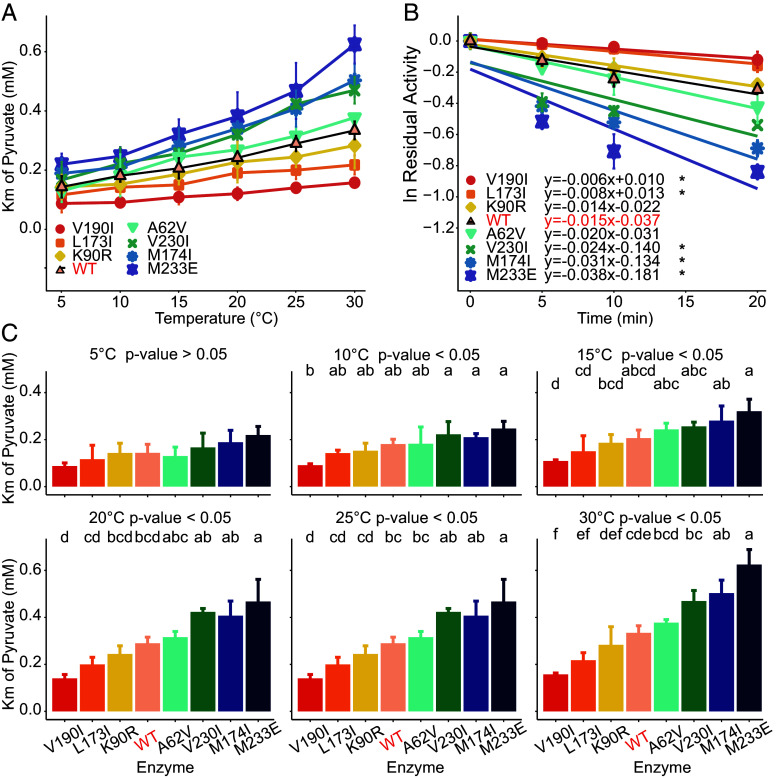
Functional validation of LDH-A amino acid sequence changes at TRSS by SDM experiments. (*A*) *K_m_^PYR^* values (mean ± SD, n = 3) of 7 single mutants and one wild-type (WT) LDH-A of zebrafish. (*B*) Thermal stabilities of seven single mutants and one wild-type LDH-A were determined as the residual activities following incubation at 50 °C for 0 to 20 mins (mean ± SD, n = 3). The results of covariance analyses are shown (α = 0.1). (*C*) *K_m_^PYR^* for LDH-A at different temperatures. Substrate reaction curves were determined at 5, 10, 15, 20, 25, and 30 °C for 7 single mutants and one wild-type LDH-A of zebrafish. ANOVAs, followed by post hoc comparisons using the LSD test, were performed on each temperature. The same letters annotated in the top indicate there was no significant difference between the mutants.

An important kinetic property of LDH-A was examined to confirm the effect of the observed TRSS amino acid substitutions on thermal adaptation of enzyme function. The Michaelis–Menten constant of pyruvate (*K_m_^PYR^*), which is strongly dependent on the temperature of measurement and known to display strong temperature-adaptive variation among species (8), was the trait used in these comparisons. At a common temperature of measurement, *K_m_^PYR^*values commonly are inversely proportional to evolutionary adaptation temperature. Our site-directed-mutagenesis-generated enzymes reflect this pattern. Thus, the cold-adapted variants exhibited higher *K_m_^PYR^* at 5, 10, 15, 20, 25, 30 °C than warm-adapted variants and the difference was especially pronounced at higher measured temperature (Fig. 6*A*). Five variants showed significant differences at 30 °C: L173I (*P*-value = 0.050), M174I (0.010), V190I (0.001), V230I (0.012), and M233E (0.002) (Fig. 6*C*). These variants also exhibited significant differences [analysis of covariance (*P*-value < 0.1)] in thermal stability measured as residual activity of the enzyme after incubation at high temperature (50 °C) for different times (Fig. 6*B*). Because of the demonstrated temperature-adaptive differences among these site-directed-mutagenesis generated LDH-As, these five substitution sites were defined as the “key” TRSS among the TRSS we validated using SDM. It is notable that the properties of hydrogen bonds and variant types (nonconservative or conservative) that significantly affect structure do not seem to invariably influence enzymatic kinetic properties. For example, although both variants A62V and V190I exhibited comparable significant differences in structural properties, only variant V190I showed significant changes in both *K_m_^PYR^* and thermal stability measurements (Figs. 5 and 6). Thus, because of their demonstrated differences in function and stability, these five key TRSS were chosen as focal points in building the following model for predicting thermal limits.

### Prediction of Species’ Thermal Limits.

**Fig. 7.**
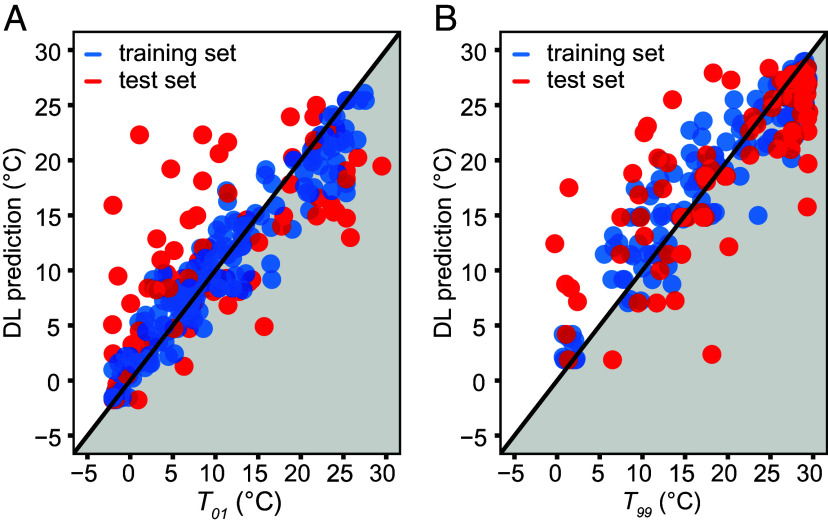
Deep learning (DL) predicted results of species’ thermal limits. (*A*) The comparison of DL prediction and physiological lower thermal limits. Lower thermal limits were defined as the 1st percentile of habitat temperatures (*T*_01_). (*B*) The comparison of DL prediction and physiological upper thermal limits. Upper thermal limits were defined as the 99th percentile of habitat temperatures (*T*_99_).

With the use of GNN and a gradient boosting model (XGBoost) framework, a deep learning model was built and trained to predict the species’ thermal limits, focusing on the key TRSS just discussed (*SI Appendix*, Fig. S1*B*). To organize the input information in the deep learning model, the structural data of the key TRSS together with the functional data (i.e., thermal adapted types of variants) validated by kinetic parameters were used to construct the model. For training, the dataset with 277 orthologs was split randomly into a training set (70%) and a test set for validation (30%). For both GNN and XGBoost, hyperparameter optimizations were selected by a fivefold cross-validation on the training set determined by the minimum mean squared error (MSE). As a result, the test set of DL for upper thermal limits prediction yields an MSE of 32.97 and an R^2^ of 0.58, while DL for lower thermal limits prediction achieves an MSE of 41.32 and an R^2^ of 0.44 (Fig. 7).

## Discussion

A few variants at a small number of substitution sites (TRSS) can lead to significant changes in enzymic structural flexibility and kinetic properties. This pattern of thermal adaptation in enzyme structure and kinetics is manifested across evolutionarily divergent lineages, demonstrating convergent evolution among species adapting to distinct thermal environments. Building on these mechanistic insights, we developed a deep learning model to predict species’ thermal tolerance limits using data on structural properties and kinetic parameters. This model may prove useful in predicting the effects of changes in temperature on distribution patterns and find other applications in resource management, fisheries, and conservation efforts where thermal responses of animals are important.

### Variants in TRSS Can Result in Thermal Adaptation of LDH-A.

A few amino acid sequence changes outside the active site can lead to striking differences in kinetic properties and structural stability. In the present study, 127 substitution sites from all 331- or 332-residue LDH-A sequences of 277 fish species whose habitat temperatures ranged from −1.6 to 29.5 °C were analyzed in order to determine whether a general mechanism (sequence sites of adaptive change (TRSS) and the nature of the amino acid changes (variants) at these sites) of LDH-A thermal adaptation occurred for fish. Numerous studies using a limited number of species have illustrated the relationship between enzyme thermal adaptation and enzyme structural stability (3, 4, 6, 7). Here, examination of a large number (277) of species enabled the identification of more comprehensive candidate substitutions and allowed statistical analyses to reveal general patterns across taxonomically diverse fish species from widely different environments.

Several general patterns were discovered that shed light on structure–function relationships in thermal adaptation. Hydrophobic interactions of amino acid residues in important secondary structures outside the active site can notably affect the enzyme’s structural and functional features. As is known in catalysis of LDH-A, conformational changes during substrate binding may be the rate-limiting steps compared with the hydride transfer that occurs during catalysis (27, 28). The conformation of LDH-A undergoes a significant change, causing a surface loop to shift and cover the active site. This loop movement is transmitted outward through hydrophobic interactions, affecting structures that do not directly interact with the ligands (28). In our results of SDM studies with zebrafish LDH-A, all the key TRSS showed significantly stronger hydrophobic interactions characteristic of warm-adapted amino acids in cases where significantly lower *K_m_^PYR^* at 30 °C were found (Fig. 6*A*). This result is consistent with previous studies of orthologs of LDH-A from warm-adapted species (6) and of orthologs of a closely related enzyme, malate dehydrogenase (15). Furthermore, given the highly conserved crystallographic structures and active site residues of LDH-A from divergent species, from bacteria to dogfish (3), the sequence changes governing adaptive shifts in kinetic properties are unlikely to occur by modification of residues involved in the direct interactions between the enzyme and substrates. The five key TRSS were distributed either within secondary structure found in proximity to the active site or on MRs. However, the other two TRSS (A62V and K90R) that had no significant effects on kinetic properties were distributed on secondary structures far from the catalytic pocket. Therefore, the most important sites of adaptation in function may be in regions such as the five key TRSS that govern the ability of catalytically important structures to alter conformation during binding and catalysis (5). Other substitutions may influence overall structural stability, e.g., as indicated by ΔRMSD values (Fig. 4*A*), without having effects on binding or catalysis. It should be noted that, despite our emphasis on a relatively small number of TRSS where adaptive change is especially common among species and often reflects convergent evolution, we recognize and in fact have discovered that amino acid substitutions can occur outside of the TRSS regions and lead to thermal adaptation in many individual cases (6, 21).

Enzyme thermal adaptation depends more on enzyme structural stability than on the use of particular amino acids, i.e., a given amino acid may play roles in adaptation to both heat and cold, depending on where the amino acid occurs in the protein and the role it plays in stabilizing structure. Previous studies have established linkages between amino acid changes at specific sites and alterations in kinetics and stability (9, 10, 25), but a general understanding of how a particular type of amino acid at a certain site in the protein contributes to temperature adaptation has been lacking. In the present study, valine of site 190 is regarded as a cold-adapted residue, and leucine of site 190 is considered a warm-adapted residue, while for site 230, it is just the opposite (i.e., leucine is a cold-adapted residue and valine is a warm-adapted residue). Although both valine and leucine are classed as hydrophobic amino acids, a significantly stronger hydrophobicity of warm-adapted residues is detected in both sites 190 and 230 by significance tests of values of SASA and RSA (Fig. 5), consistent with the important roles that hydrophobic stabilization plays in stabilizing enzyme structure. As mentioned above, differential, site-specific effects for a given type of residue are common in proteins, as the effects of the residues on protein stability and function are shaped by both their physicochemical properties and the characteristics of neighboring residues (e.g., size, polarity, charge, hydrophobicity).

### Convergent Evolution in Enzyme Thermal Adaptation.

Similar patterns of adaptation found in this large-scale evaluation of LDH-A of fish that have diverged over 200 MA provide a clear example of convergent evolution in enzyme thermal adaptation. The TRSS we found in our analyses of phylogenetically diverse fishes reflect widespread evidence for convergent evolution (4, 29). Thus, these TRSS were all identified as common sites of variation in amino acid composition that were statistically significantly linked to variation in adaptation temperature. These statistically identified residues were then examined (“validated”) with two further types of experiments to more directly assess their adaptive roles. In silico and in vitro experiments showed the adaptive structural and functional consequences of amino acid substitutions at these critically important sites. Previous studies have reported similar convergent evolution in LDH-As, such as the instance of amino acid substitution shared at position 310 within helices αH (5, 6, 10). In the present study, eighteen TRSS (including the five key TRSS) with the characteristics of significant hydrophobicity change and important secondary structure roles were analyzed. The demonstration of taxonomically widely distributed TRSS and evidence of convergent evolution in LDH-As offers a basis for predicting the thermal limits and distribution of species based on biochemical information.

### Application of Biochemical Traits in Identifying Species’ Thermal Limits.

Deep learning based on a large number (277 in the present study) of taxa facilitates the prediction of species’ thermal limits based on a few key TRSS. Based on biochemical data, including structural data (SASA, RSA, number, and distance of hydrogen bonds for each key TRSS) and functional data (thermal-adapted SDM-generated variant types for key TRSS), the framework of GNN and XGBoost performs well on simulating species upper and lower thermal limit temperatures on the test set. As an impactful method to extract features from molecular data, GNN fingerprints are widely used in task-specific deep learning to help molecular representation (30). Previous studies have proven the superiority of the joint effect of GNN and XGBoost in regression forecasting compared with the use of only one of the algorithms; the joint method is also widely used in predicting enzymes’ kinetic parameters (18, 31). Therefore, with the rapidly developing sequencing technology and well-performing deep learning frameworks, a tractable way to predict species’ thermal limits is established. This approach would find especially great utility for species lacking extensive physiological and distributional data and could prove especially important in the cases of economically important and protected species. The simulated thermal limits can also be used to predict species distribution ranges in current conditions and under scenarios for future changes in climate.

## Materials and Methods

### LDH-A Sequence Preparation.

We compiled 1531 genomes of fishes, including representatives from the classes Chondrichthyes, Cyclostomata, and Osteichthyes, from the National Center for Biotechnology Information Assembly database (NCBI, https://www.ncbi.nlm.nih.gov/). To extract the orthologs of LDH-A from the genome data, we mapped the LDH-A sequence to the large set of genomes by using the BLAST-Like Alignment Tool (*BLAT*) (32), followed by the extraction of the located scaffolds. Then, the *GenomeThreader* was used to generate homology-based LDH-A prediction with the combined coding DNA sequence (CDS) of predicted exon regions. CDSs were translated to protein sequences using a tritiered qualified inspected approach, which include filtering out sequences that had one or more of the following characteristics: 1) a stop codon was not present at the end of the protein sequence; 2) a stop codon or unknown codon (appeared as amino acid *X* using translated software *SeqKit v2.2.0*) appeared in the middle of the protein sequence; and 3) no matches or percentage of identical matches (pident) < 90 occurred when using *BLASTp v2.13.0 +* to filter sequences.

The LDH-A sequence dataset was further compiled with an additional 1903 LDH-A sequences extracted from the NCBI Nucleotide database. We further restricted the sequence length to 331 or 332 amino acids (5, 6). Only one deduced amino acid sequence was retained for each species by considering the genome assembly quality based on assembly level (i.e., Chromosome > Scaffold > Contig), contig N50 (i.e., the sequence length of the shortest contig at 50% of the total assembly length), and scaffold N50 (genome information in Dataset S1). As some species shared the same deduced amino acid sequence, a total of 197 isoforms from 277 fish orthologs were gathered. Sequences were aligned by using *MAFFT v7.487* (33), and the sequence logos were generated by aligning LDH-A sequences to the *ggseqlogo v0.2 R* package (34).

### Species Distribution and Historical Habitat Temperature.

Information on fish habitats and life history was gathered from FishBase (https://fishbase.mnhn.fr/search.php). Based on this information, a large set of marine fishes were selected for our analyses. We downloaded fish occurrence data, including longitudes, latitudes, and water depths, from Global Biodiversity Information Facility (GBIF, https://www.gbif.org/) and Ocean Biodiversity Information System (OBIS, https://obis.org/) using *R v4.2.3*. We downloaded the World Ocean Atlas in 2018 (WOA2018) with a 0.25 × 0.25 arcdegree resolution for averaged decades from 1955 to 2017. Furthermore, we extracted species habitat temperature from the WOA2018 environmental data depending on species distribution of longitude, latitude, and depth (Fig. 1*A*).

### Taxonomy and Phylogeny Construction.

We collected information about fish taxonomy from databases of NCBI, GBIF, and related literature (Fig. 1*B*). Phylogenetic relationships and estimated divergence times among all known classes in our study were built based on the comprehensive phylogeny of ray-finned fishes (35).

### TRSS Detection.

For detecting TRSS, our overall strategy involved sequence alignment, binomial regressions, and significance tests (*SI Appendix*, Fig. S1*A*). Here, we use the term “substitution site” to refer to sites in the sequence that contain different amino acids in species adapted to different temperatures. We refer to these different amino acids at substitution sites as “variants” (5, 7). We utilized the following protocols to detect which substitution sites fit our definition of TRSS: 1) We initially filtered variants of each substitution site. For each substitution, only variants with a frequency greater than one (n ≥ 2) were gathered. A total of 365 variants among the 127 substitution sites were used in the following analyses. 2) We performed binomial regression analyses between variant probability and species’ average habitat temperature. For each substitution, binomial regressions were conducted using *R v4.2.3*. 3) We performed significance tests on the species’ average habitat temperature between different variants at each substitution site. Tests for significant differences were performed using ANOVA, followed by post hoc comparisons using the LSD test for the parameter test or the Kruskal–Wallis test, followed by the Dunn multiple comparisons test for the nonparametric test, with a Bonferroni adjustment at α = 0.05, in *R v4.2.3*. As a result, substitution sites defined as TRSS should fit the following three criteria: 1) TRSS should have more than one (n ≥ 2) variant to avoid effects of sequencing mistakes. 2) TRSS should have variants with a *P*-value below 0.0001 in the binomial regressions. 3) TRSS should have variants with a *P*-value below 0.0001 in the significance tests. For each TRSS, variants in LDH-A from species with significantly lower average habitat temperature from the significance tests described above were defined as a cold-adapted variant type, while variants with significantly higher average habitat temperature from the significance tests described above were defined as a warm-adapted variant type.

### Protein Structure Modeling and Comparison.

Recently developed computational tools for predicting key elements of protein structure can yield new insights into protein structure–function relationships and the mechanistic bases of adaptive changes in sequence. To this end, we utilized the *AlphaFold*-based workflow provided by *ColabFold v1.5* to predict 3D structures of LDH-As (19). By integrating the MMseqs2 algorithm, *ColabFold* enables large-scale predictions with approximately a 90-fold speed improvement and higher prediction quality compared to the original *AlphaFold2*. Here, we performed five repeated models (--num-models) for each LDH-A sequence based on the custom templates of 5 fish LDH-A 3D structures (PDB accession number: 1ldm, 2v65, 3ldh, 6ldh, and 8ldh) with 3 prediction cycles (--num-recycles) and structural relaxation (--amber). The models having the highest energy minimization scores among the 5 repetitions were used for further analyses.

A series of structure metrics, including number of hydrogen bonds, distance of hydrogen bonds, SASA, and RSA, were calculated to elucidate enzymes’ properties in conducting structural comparisons. Hydrogen bonds, which were identified as polar contacts in proteins, were analyzed by both number and distance by the *VMD* program (36). Hydrogen bonds are one of the main factors contributing to protein stability (3, 11, 37). Larger numbers and shorter distances of hydrogen bonds indicate stronger interactions, resulting in greater protein stability. SASA, which was measured as the contact area between molecule and solvent, was computed by the program *DSSP* on the Linux platform (38) and RSA was calculated by the equation $RSA=\frac{\mathrm{SASA}}{\mathrm{MaximumSASA}}$ (39). Both SASA and RSA reveal the solvent exposure of amino acids and are correlated with protein hydrophobicity (26). Protein hydrophobicity has commonly been studied in enzymatic thermal adaptation because hydrophobicity is strongly associated with changes in entropy of the enzyme and surrounding solvent during folding and unfolding (5, 40). As a result, smaller values in SASA and RSA indicate stronger hydrophobicity, which contributes to increased protein stability, especially at high temperatures. Tests for significant differences among variants at each TRSS were conducted using ANOVA followed by LSD post hoc comparisons for the parameter test, or the Kruskal–Wallis test followed by the Dunn multiple comparisons test for the nonparametric test, with a Bonferroni adjustment at α = 0.05, in *R v4.2.3*.

### Molecular Dynamic Simulation.

In silico molecular dynamics studies can yield deep insights into the structural flexibility of the protein backbone and the movements of each residue in the sequence. Reversible movements of catalytically important regions of an enzyme’s structure are critical for function and play important roles in temperature adaptation (15, 25). Forty-five species of fish, with different habitat temperatures, were selected for molecular dynamics simulations. These forty-five LDH-A orthologs can be categorized into eight isoforms based on the identical deduced amino acid sequences (*SI Appendix*, Table S1). Molecular dynamics were calculated in *Gromacs v2021.3* (41) using the OPLS-AA/M force field (7). A transferable intermolecular potential tip3p water model with a cubic system was used to create a cellular-like environment. These solvated systems further underwent energy minimization to eliminate energetically unfavorable interactions between ions and water molecules (50,000 steps). Quintic simulations for each isoform were performed five times for 20 ns in the isobaric-isothermal condition at 10 and 30 °C by velocity rescale thermostat and 1 bar pressure by the Parrinello–Rahman method (42). Trajectories of the structures were recorded and saved at intervals of 0.002 ns throughout the simulation.

For all trajectories, the RMSD of the backbone and the RMSF of each residue for the stabilized stage (10 to 20 ns) were calculated (25). They are defined as $RMSD=\sqrt{\frac{1}{N}\sum_{i=1}^{N}{\left(r_{i}-r_{\mathrm{ref}}\right)}^{2}}$ and $RMSF=\sqrt{\frac{1}{T}\sum_{t=1}^{T}{\left(r_{t}-r_{\mathrm{ref}}\right)}^{2}}$, where *r_ref_* represents the reference position of an atom, *r_i_* represents the position at atom *i*, *r_t_* represents the position at time *t*, *N* represents the total number of atoms, and *T* represents the total time of calculating *RMSF*. Differences between the values obtained at 10 and 30 °C for RMSD (ΔRMSD) were used to estimate the overall protein backbone flexibility; a high value of ΔRMSD indicates high protein flexibility. To determine how ΔRMSD varied with evolutionary adaptation temperature, a linear regression analysis was performed between ΔRMSD and the average temperature encountered by the species in its habitat, using *R v4.2.3.*

### SDM and Catalytic Functional Validation.

Zebrafish, as a genetically tractable model species of fish, was used to validate the functional significance of several identified TRSS (43, 44). The clone CDS (NCBI: BC165309.1) of zebrafish LDH-A was synthesized directly (Sangon Biotech, Shanghai, China) and used as the platform for SDM. Among 18 TRSS, substitutions that satisfied the following requirements were selected to further explore the mechanistic linkages between enzyme structure and function: 1) stronger hydrophobicity characteristics were exhibited in warm-adapted variant types compared to cold-adapted variant types; 2) substitutions were located within secondary structure in proximity to active sites or MRs (3, 6). To figure out the general pattern, we focused on hydrophobic characteristics because hydrophobic interactions play an important role in maintaining protein 3D structure (45). Furthermore, hydrophobic interactions in enzymatic thermal adaptation have been widely studied and the findings consistently show that warm-adapted enzymes exhibit more hydrophobic interactions (8). In addition, earlier studies also indicated that substitutions near the important secondary structures that alter conformation during function may result in enzymatic thermal adaptation (5). Following these selection criteria, seven TRSS were used in SDM experiments. Considering that the sequence length of zebrafish LDH-A is 333, which is different from 331/332 of the marine fishes in our study, we first aligned all sequences using *MEGA11* (46). After alignment, seven TRSS (62#, 90#, 173#, 174#, 190#, 230#, and 233#; numbered according to the 332 residue sequences), which were localized in different secondary structures and MRs, were used in SDM to generate both conservative and nonconservative variants (Sangon Biotech, Shanghai, China). In these experiments, four mutagenesis-generated changes (A62V, M174I, V230I, M233E) were created to examine an adaptive change from warm- to cold temperatures; the other three changes (K90R, L173I, V190I) were made to examine adaptive shifts from cold- to warm temperatures.

Vectors pET32a(+) ligated with one of the eight LDH-A nucleotide sequences were transformed into the expression host BL21(DE3) competent cells (Sangon Biotech, Shanghai, China). Following that, they were plated on Luria-Bertani (LB) agar medium with 100 μg/mL ampicillin and incubated overnight at 37 °C in a shaking incubator (Zhichu, Shanghai, China; ZQZY-78BN). A single clone of each plate used for further expression was validated by sequencing with common primers of pET32a(+) (T7-TER and S-TAG). These clones were inoculated into 500 mL LB/ampicillin broth separately and incubated with constant 180 rpm shaking at 37 °C for ~5 h until growth reached the log phase. Expression was induced in BL21(DE3) with 1 mmol/L isopropyl-β-D-thiogalactopyranoside (IPTG). Culturing of the cells was continued for another 4 h at 30 °C, with shaking at 220 rpm; afterward, the pelleted cells were collected by centrifugation (4 °C) at 8,000 rpm for 10 min.

Extraction and purification of the target expressed protein were conducted using ultrasonic disruption and His-tag affinity purification. The sedimented cells were resuspended in 30 mL lysis buffer (50 mmol/L potassium phosphate, pH 6.8, 0.1% Triton X-100, and 1 mmol/L PMSF), following by cell disruption performed on an ultrasonic cell disruptor with 30% amplitude (5 s disruption and 5 s rest) for 30 min, during which an ice water mixture was used to maintain a low temperature. The supernatant was collected after centrifugation at 4 °C and 9,500 rpm for 1.5 h and was further purified through a 10-mL Ni-NTA agarose column (Sangon Biotech, Shanghai, China). Following the manufacturer’s instructions, after the column was balanced with 20 mL lysis buffer (10 mmol/L imidazole, pH 7.4), 20 mL supernatant was passed over the column twice to bind the poly-His-tag to the column tightly. In order to wash out other proteins, 20 mL of washing buffer (20 mmol/L imidazole, pH 7.4) was used to wash the column twice. After these washings, the purified LDH-A protein was eluted with 20 mL elution buffer (250 mmol/L imidazole, pH 7.4) and dialyzed against potassium phosphate buffer (50 mmol/L potassium phosphate, pH 6.8) overnight. All steps in the purification were conducted at 4 °C.

Kinetics measurements using the purified and dialyzed enzymes were performed on a Shimadzu UV-1900i UV-Visible spectrophotometer (Shimadzu, Kyoto, Japan) with a water bath (Tianheng, Ningbo, China) for temperature control. For each assay, conversion of NADH to NAD^+^ during the reaction was monitored at 340 nm. For determining *K_m_* of pyruvate (*K_m_^PYR^*), the assay medium contained imidazole-Cl buffer (80 mmol/L, pH 7.0, at 20 °C), NADH (150 μmol/L), and sodium pyruvate (0.1, 0.2, 0.4, 0.6, 0.8, and 1 mmol/L). Substrate reaction curves were derived at 5, 10, 15, 20, 25, and 30 °C, and *K_m_^PYR^* values were calculated with nonlinear regression analysis using *Prism 9.5* software (9). Thermal stability of LDH-A was measured by determining the residual enzyme activity at 20 °C after incubation at 50°C for 5, 10, and 20 min, as described by ref. 5. Covariance analyses for residual activity were conducted on *SPSS* (IBM SPSS Statistics 25) using incubation time as the covariate. As a result of these in vitro experiments, among the seven validated TRSS (62#, 90#, 173#, 174#, 190#, 230#, and 233#), we identified a total of five key TRSS, defined as TRSS of SDM-generated enzymes that showed significant differences in *K_m_^PYR^* and thermal stability compared to the wild type.

### Deep Learning for Simulating Species’ Thermal Limits.

Molecular structural features, including the number of hydrogen bonds, distances of hydrogen bonds, SASA, and RSA, were integrated by the graph neural network (GNN) using the deep learning library *TensorFlow* (*SI Appendix*, Fig. S1*B*) (47). These molecular structural features for the five key TRSS defined above were regularized into an (*5 × 4*)-dimensional tensor. Arranging in sequence order, a (*5 × 5*)-dimensional tensor of the symmetric normalized Laplacian matrix was used to update the feature vectors of nodes and edges during the iteration process. Nonlinearities were introduced by the activation function *ReLU*. Global average pooling of *GlobalAveragePooling1D* was used to extract the GNN-pooled fingerprint vectors with the goal of predicting the species’ thermal limit temperature.

Following creation of the GNN model, a gradient-boosting library, XGBoost, was added to simulate species’ thermal limits further. The structural data, derived from the GNN fingerprints, and the functional data, based on the thermal-adapted type of residues, were jointly utilized to train a gradient boosting model. Upper thermal limits were considered as the 99th percentile of habitat temperatures (*T*_99_), while lower thermal limits were defined as the 1st percentile of habitat temperatures (*T*_01_) (48).

Hyperparameter optimization plays an important role in training deep learning models (49). All hyperparameter optimizations were performed on a 5-fold cross-validation on the training set and selected by the MSE. Batch size, learning rate, and the epochs for the GNN model were trained, and learning rate, maximum delta step, maximal tree depth, hyperparameter regularization coefficients, number of training rounds, and minimum child weight were optimized for the gradient boosting model by performing a grid search. Additionally, all the data preparation was organized on *R v4.2.3*, and models were built and trained on *Python v3.8.20*.

## Supplementary Material

Appendix 01 (PDF)

Dataset S01 (XLSX)

## Data Availability

All study data are included in the article and/or supporting information.
