# Supplementary material for: Temperature adaptation in structure and function in lactate dehydrogenase-A reflects convergent evolution in a few key protein regions
Source: Proc Natl Acad Sci U S A. 2025 Oct 10;122(42):e2517759122. doi: 10.1073/pnas.2517759122 (PMC12557798; doi:10.1073/pnas.2517759122)
Supplement: Supplementary file 1 — Appendix 01 (PDF) [file pnas.2517759122.sapp.pdf]

PNAS

Supporting Information for

Temperature adaptation in structure and function in lactate dehydrogenase-A reflects convergent evolution in a few key protein regions

Xiao-Lu Zhu[a,b,c], Ming-Ling Liao[a,b], Lin-Xuan Ma[a,b], George N. Somero[d], Yun-Wei Dong[a,b,1]

[a]The Key Laboratory of Mariculture, Ministry of Education, Fisheries College, Ocean University of China, Qingdao, 266003, China
[b]Shandong Key Laboratory of Green Mariculture and Smart Fishery, Fisheries College, Ocean University of China, Qingdao, 266003, China
[c]Academy of Future Ocean, Ocean University of China, Qingdao, 266100, China
[d]Department of Biology, Hopkins Marine Station, Stanford University, Pacific Grove, CA 93950

[1]To whom correspondence may be addressed: Yun-Wei Dong
Email: dongyw@ouc.edu.cn

This PDF file includes:

Figures S1 to S2
Tables S1 to S2

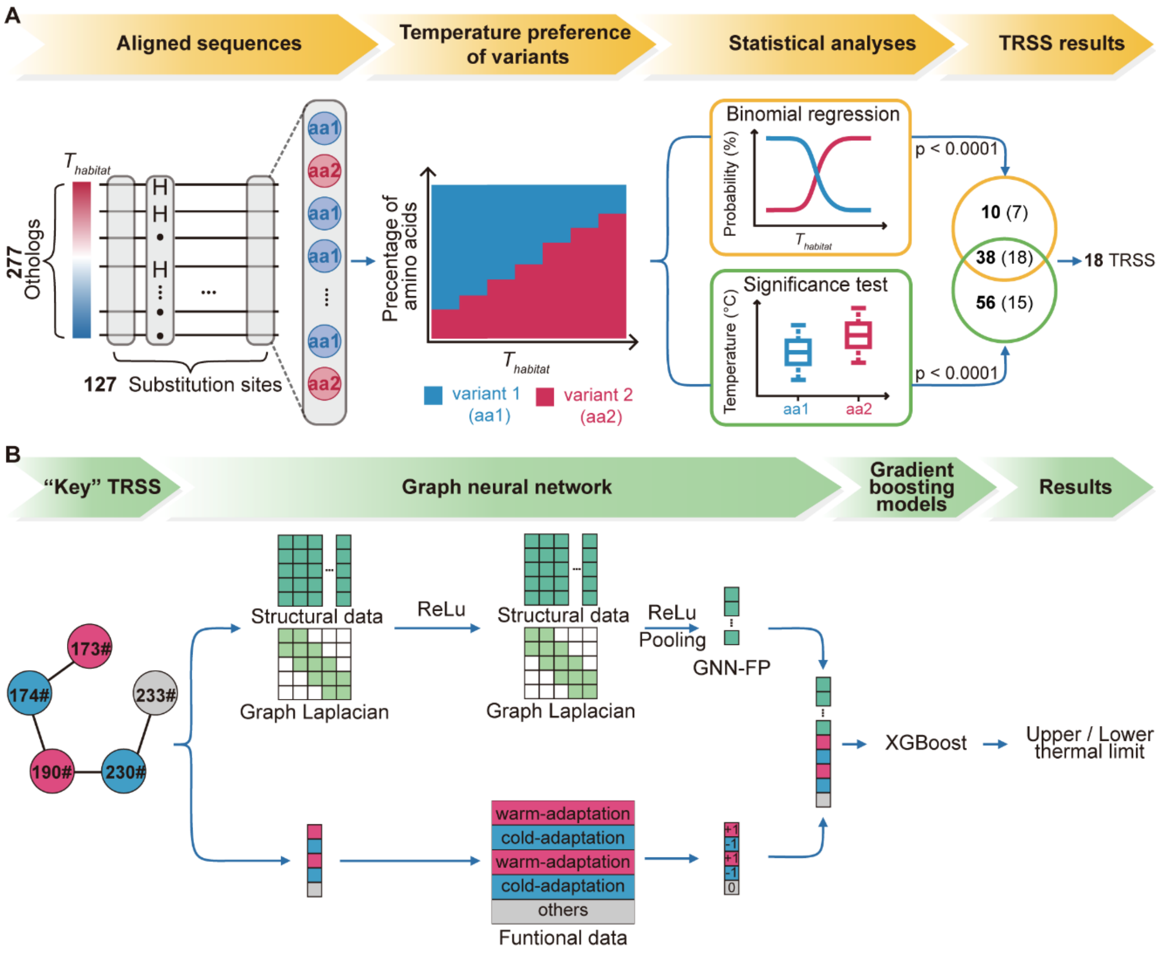

Figure S1. General workflow of the present study. (*A*) Overview of the approach for TRSS detection. The preference of amino acids in orthologs from different habitat temperatures was detected after the alignment of 277 orthologs. The dot represents the alignment gap of LDH-As at site 75. Binomial regression and significance tests were performed on 127 substitution sites. Only the substitutions with variants showing a significance (p-value < 0.0001) were considered as TRSS. TRSS results given in a Venn plot show the variant number in bold type and substitution site number in parentheses. (*B*) Biochemical trait-based deep learning model of species' thermal limits. Node and edge feature vectors were iteratively updated and pooled together as the graph neural network fingerprints (GNN-FP). Connected with adaptational information, upper and lower thermal limits were predicted through the gradient boosting model.

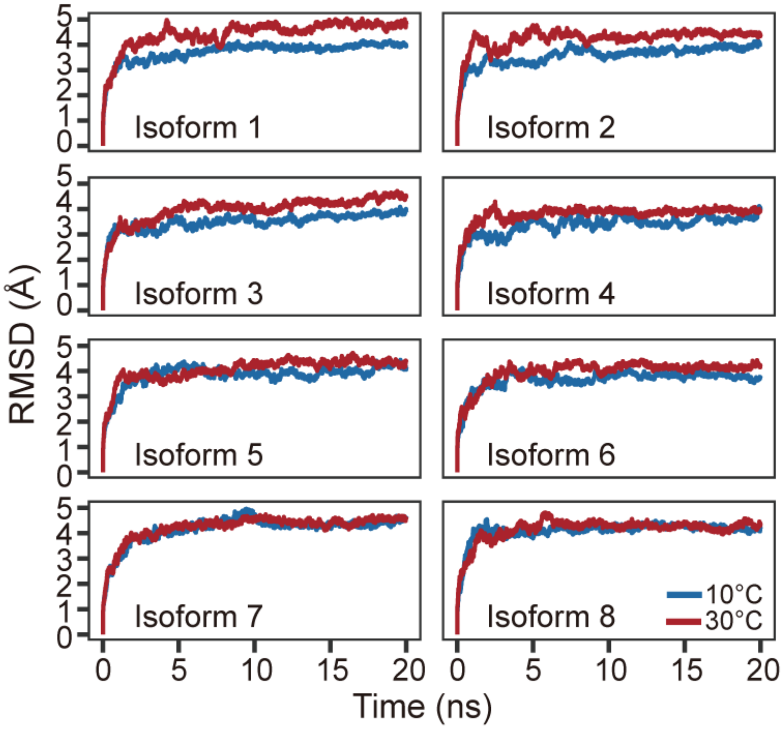

Figure S2. The root mean square deviation (RMSD) of backbone atom positions for LHD-As at 10°C and 30°C. Molecular dynamics simulation analyses were performed on eight different isoforms of LDH-A (Supplementary Table S1).

Table S1. Information about isoforms used in molecular dynamic simulation.

Isoform	Isoform average temperature (°C)[1]	Subclass	Order	Family	Species	Species average habitat temperature (°C)
Isoform1	3.73	Actinopteri	Perciformes	Sebastidae	*Sebastes ciliatus*	4.80
Isoform1	3.73	Actinopteri	Perciformes	Sebastidae	*Sebastes minor*	1.68
Isoform1	3.73	Actinopteri	Perciformes	Sebastidae	*Sebastes variabilis*	4.72
Isoform2	10.87	Actinopteri	Perciformes	Sebastidae	*Sebastes aleutianus*	5.17
Isoform2	10.87	Actinopteri	Perciformes	Sebastidae	*Sebastes atrovirens*	14.82
Isoform2	10.87	Actinopteri	Perciformes	Sebastidae	*Sebastes auriculatus*	13.34
Isoform2	10.87	Actinopteri	Perciformes	Sebastidae	*Sebastes babcocki*	8.05
Isoform2	10.87	Actinopteri	Perciformes	Sebastidae	*Sebastes carnatus*	14.68
Isoform2	10.87	Actinopteri	Perciformes	Sebastidae	*Sebastes caurinus*	13.36
Isoform2	10.87	Actinopteri	Perciformes	Sebastidae	*Sebastes crameri*	9.07
Isoform2	10.87	Actinopteri	Perciformes	Sebastidae	*Sebastes dallii*	12.09
Isoform2	10.87	Actinopteri	Perciformes	Sebastidae	*Sebastes diploproa*	9.41
Isoform2	10.87	Actinopteri	Perciformes	Sebastidae	*Sebastes flavidus*	8.84
Isoform2	10.87	Actinopteri	Perciformes	Sebastidae	*Sebastes goodei*	11.87
Isoform2	10.87	Actinopteri	Perciformes	Sebastidae	*Sebastes hopkinsi*	12.08
Isoform2	10.87	Actinopteri	Perciformes	Sebastidae	*Sebastes itinus*	7.47
Isoform2	10.87	Actinopteri	Perciformes	Sebastidae	*Sebastes levis*	12.05
Isoform2	10.87	Actinopteri	Perciformes	Sebastidae	*Sebastes maliger*	8.20
Isoform2	10.87	Actinopteri	Perciformes	Sebastidae	*Sebastes melanops*	12.62
Isoform2	10.87	Actinopteri	Perciformes	Sebastidae	*Sebastes melanostomus*	11.97
Isoform2	10.87	Actinopteri	Perciformes	Sebastidae	*Sebastes miniatus*	12.79
Isoform2	10.87	Actinopteri	Perciformes	Sebastidae	*Sebastes moseri*	9.35
Isoform2	10.87	Actinopteri	Perciformes	Sebastidae	*Sebastes nebulosus*	13.79
Isoform2	10.87	Actinopteri	Perciformes	Sebastidae	*Sebastes nigrocinctus*	7.92
Isoform2	10.87	Actinopteri	Perciformes	Sebastidae	*Sebastes pinniger*	9.16
Isoform2	10.87	Actinopteri	Perciformes	Sebastidae	*Sebastes rastrelliger*	13.33
Isoform2	10.87	Actinopteri	Perciformes	Sebastidae	*Sebastes reedi*	6.38
Isoform2	10.87	Actinopteri	Perciformes	Sebastidae	*Sebastes rubrivinctus*	10.40
Isoform2	10.87	Actinopteri	Perciformes	Sebastidae	*Sebastes semicinctus*	12.11
Isoform2	10.87	Actinopteri	Perciformes	Sebastidae	*Sebastes serriceps*	13.11
Isoform3	15.06	Actinopteri	Syngnathiformes	Syngnathidae	*Hippocampus abdominalis*	15.06
Isoform4	15.39	Actinopteri	Perciformes	Sebastidae	*Sebastes hubbsi*	18.78
Isoform4	15.39	Actinopteri	Perciformes	Sebastidae	*Sebastes inermis*	14.51
Isoform4	15.39	Actinopteri	Perciformes	Sebastidae	*Sebastes joyneri*	13.32
Isoform4	15.39	Actinopteri	Perciformes	Sebastidae	*Sebastes koreanus*	12.40
Isoform4	15.39	Actinopteri	Perciformes	Sebastidae	*Sebastes nivosus*	16.92
Isoform4	15.39	Actinopteri	Perciformes	Sebastidae	*Sebastes oblongus*	17.98
Isoform4	15.39	Actinopteri	Perciformes	Sebastidae	*Sebastes thompsoni*	13.81
Isoform5	17.57	Actinopteri	Spariformes	Sparidae	*Diplodus sargus*	17.57
Isoform6	21.68	Actinopteri	Syngnathiformes	Syngnathidae	*Hippocampus whitei*	21.68
Isoform7	26.43	Actinopteri	Perciformes	Serranidae	*Cromileptes altivelis*	26.41
Isoform7	26.43	Actinopteri	Perciformes	Serranidae	*Epinephelus coioides*	25.86
Isoform7	26.43	Actinopteri	Perciformes	Serranidae	*Epinephelus fuscoguttatus*	27.28
Isoform7	26.43	Actinopteri	Perciformes	Serranidae	*Epinephelus lanceolatus*	26.16
Isoform8	28.26	Actinopteri	Syngnathiformes	Syngnathidae	*Hippocampus comes*	28.26

[1] Isoform average temperature was calculated as the average temperature of the species' average habitat temperature. Many species had the same protein isoform.

Dataset S1. Information of species name, NCBI ID, genomic information, taxonomy data, deduced amino acid sequences and mean habitat temperature.

See the separate file (Dataset S1.xlsx).
